# Polymer Conjugates of Antimicrobial Peptides (AMPs) with d-Amino Acids (d-aa): State of the Art and Future Opportunities

**DOI:** 10.3390/pharmaceutics14020446

**Published:** 2022-02-19

**Authors:** Ottavia Bellotto, Sabrina Semeraro, Antonella Bandiera, Federica Tramer, Nicola Pavan, Silvia Marchesan

**Affiliations:** 1 Chemical and Pharmaceutical Sciences Department, University of Trieste, 34127 Trieste, Italy; ottavia.bellotto@phd.units.it (O.B.); ssemeraro@units.it (S.S.); 2 Life Sciences Department, University of Trieste, 34127 Trieste, Italy; abandiera@units.it (A.B.); ftramer@units.it (F.T.); 3 Medical, Surgical and Health Sciences Department, University of Trieste, 34127 Trieste, Italy; npavan@units.it

**Keywords:** d-amino acids, antimicrobial, peptides, polymer, conjugate, biomaterials, resistance, gels, covalent, coatings

## Abstract

In recent years, antimicrobial peptides (AMPs) have enjoyed a renaissance, as the world is currently facing an emergency in terms of severe infections that evade antibiotics’ treatment. This is due to the increasing emergence and spread of resistance mechanisms. Covalent conjugation with polymers is an interesting strategy to modulate the pharmacokinetic profile of AMPs and enhance their biocompatibility profile. It can also be an effective approach to develop active coatings for medical implants and devices, and to avoid biofilm formation on their surface. In this concise review, we focus on the last 5 years’ progress in this area, pertaining in particular to AMPs that contain d-amino acids, as well as their role, and the advantages that may arise from their introduction into AMPs.

## 1. Introduction

The vast majority of pharmaceutical companies have severely decreased their R&D investment towards the development of new antimicrobial (AM) agents. This is due to the poor economical returns for antibiotics that have been approved for market over the last few decades [1]. Conversely, we are witnessing today a revival of research towards novel AM agents, because of the global emergency that we are all facing in terms of antimicrobial resistance (AMR), with the term AM referring to antibiotics, anti-viral and anti-malarial agents. There are various causes for the spread of AMR, comprising first and foremost the well-known misuse of AM agents to treat infections both in humans and animals, especially in countries with a lack of regulations on their use, and where a medical prescription is not required to access them. Furthermore, clear guidelines pertaining to the safe disposal of expired and unused antibiotics are urgently needed. This aspect is crucial to reduce the amount of AM drugs that are released into the environment, thus causing further AMR spreading. Other important factors to counter infections are good hygiene and appropriate sanitation programs. Overall, failing to address all these points leads to the concrete risk of entering the so-called “post-antibiotic” era, where even simple infections may become a significant cause of mortality worldwide [2].

**Figure 1:**
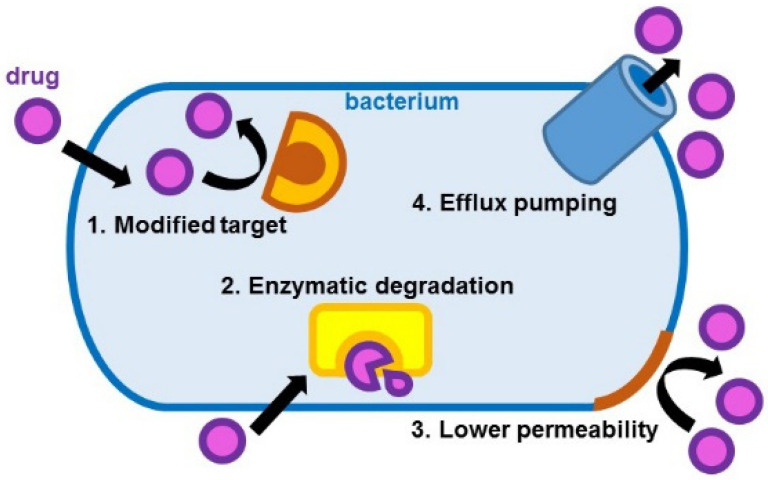
AMR emergence through four mechanisms.

Within pathogens, there are mainly four mechanisms (Figure 1) through which AMR emerges:

1. Modification of the drug target site that leads to ineffective drug binding;
2. Drug inactivation through enzymatic hydrolysis or modification;
3. Reduced drug entrance because of low permeability;
4. Increased drug elimination through efflux pumping [3].

The Centres for Disease Control and Prevention (CDC) 2019 Antibiotic-Resistance Threats Report lists multi-drug resistant (MDR) pathogens, divided into groups based on the decreasing level of emergency and severity of the required response, in the order: urgent, serious, or concerning threats, followed by a watch list [4,5]. Useful acronyms that include very dangerous MDR pathogens are ESKAPE (*E. faecium*, *S. aureus*, *K. pneumoniae*, *A. baumannii*, *P. aeruginosa* and *Enterobacteriaceae*) and ESCAPE (*E. faecium*, *S. aureus*, *C. difficile*, *A. baumannii*, *P. aeruginosa* and *Enterobacter* species). These clinically relevant microbes often possess mobile genetic elements that facilitate the spread of resistance and enable biofilm formation not only on host tissues, but also on surfaces [6]. The acronyms refer to their ability to escape the action of AM agents for which they are notorious culprits of nosocomial infections that are associated with the highest risks of mortality and healthcare elevated costs [7]. The World Health Organization (WHO) lists ESKAPE pathogens among those against which novel AMs are needed with urgency, and have further been classified into medium, high, and critical priority [8]. Therefore, it is not surprising that antimicrobial peptides (AMPs) are receiving increasing attention as alternatives to antibiotics to address these challenges, and the inclusion of d-aa is particularly relevant as an attractive strategy to overcome some of their existing limitations, as discussed further below. To the best of our knowledge, this is the first review that focuses on AMPs with d-aa that have been covalently conjugated to polymers, and it gathers the available information on the occurrence and role of d-aa to assist with the future design of enhanced AMPs, and related materials for their delivery.

### 1.1. d-Amino Acids (d-aa) in Nature

**Scheme 1:**
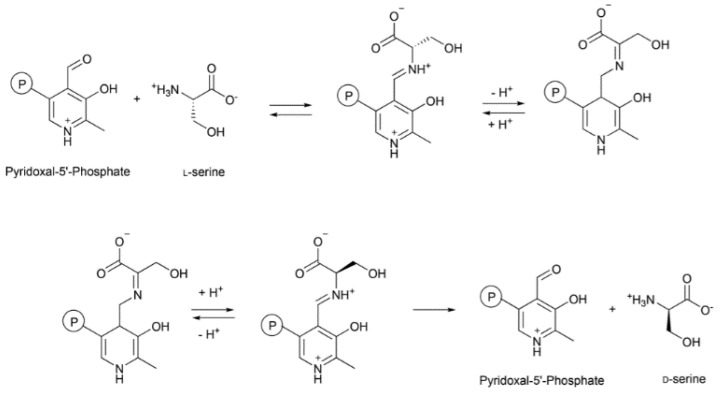
Mechanism of action of pyridoxal-5′-phosphate-dependent serine racemase. Reproduced from [13] (The Royal Society of Chemistry, 2020) upon adaption from [12], Springer, 2017.

d-aa are non-proteogenic, yet they have been found to occur in bioactive peptides in a wide variety of organisms [9], where their presence is important to increase their potency through the definition of specific conformations and increased resistance against enzymatic hydrolysis [10]. Their occurrence in metazoan organisms is well-documented, but it is anticipated that d-aa-containing peptides may have physiological effects within humans that are yet to be discovered [11]. In particular, d-Ser and d-Asp are the most abundant d-aa found in mammals, where they have physiological roles in neuromodulation and endocrine function [12]. d-aa are not genetically encoded and typically arise through post-translational isomerization, as shown in the mechanism depicted in Scheme 1.

**Figure 2:**
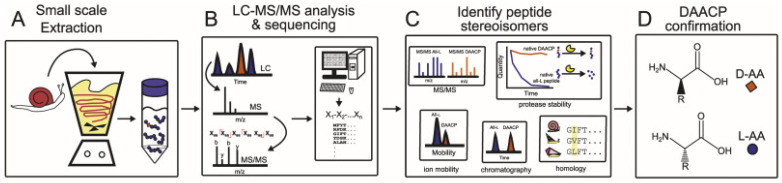
General workflow for d-aa containing peptides (DAACP). (**A**) extraction (**B**) LC-MS/MS analysis and sequencing by database search to identify native peptides. (**C**) Identification of putative isomeric peptides detected in LC-MS/MS analysis by analysing ion mobility migration, MS/MS, protease stability, chromatographic retention, or homology. (**D**) DAACP confirmation by comparison against the properties of native peptides and using synthetic standards. Reprinted with permission from Ref. [11]. Copyright 2020 Elsevier.

In terms of bioactivities, peptides with d-aa can exert neuroexcitatory [14,15] and cardioexcitatory roles [16], but also opioid [17] and anti-hypertensive [18] activity, endocrine function [19], and AM roles [20]. Peptides with d-aa have been proposed for cancer therapy [21] as adhesive biomaterials [22], vaccine adjuvants [23], and as inhibitors of pathological amyloid fibrillization [24,25,26,27,28]. Furthermore, their occurrence in human peptides within the context of disease states has been detected thanks to sensitive, modern, analytical techniques (Figure 2) [11], and it has been proposed for biomarker detection strategies for diagnostics [29,30,31,32]. The detection of d-aa nevertheless remains a challenging task due to their lower occurrence relative to l-analogues in natural samples, for which workflow optimized protocols and new methods continue to emerge [33,34,35].

How d-aa affect L-peptides is still to be fully clarified. It is well-known that they may favour turn conformations [36], and to this end the inclusion of d-aa is a documented strategy in their design [37]. However, exactly predicting which type of turn is obtained is a different matter [36], one that is further complicated by the traditional turn assignment, based also on dihedral angles as those found on L-peptides [38], and their associated common conformations based on the Ramachandran plot [39,40]. It is worth noting that even a single amino acid isomerization from L- to D- can lead to quite different physicochemical properties [11]. One is increased hydrophobicity, which can be convenient to induce hydrophobically driven self-organization into nanostructured biomaterials, for instance [26,41,42]. Remarkably, this effect is seen in sequences as short as unprotected dipeptides [43,44] without further structural modifications [45,46].

### 1.2. d-aa in Bacteria

**Figure 3:**
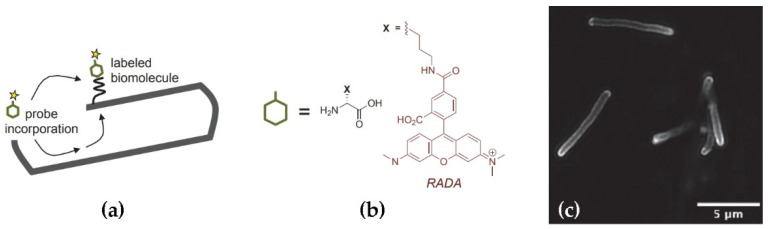
d-aa fluorescent derivatives (e.g., RADA) have been proposed to visualize peptidoglycan biosynthesis. (**a**) Schematic for bacteria metabolic labelling with RADA. (**b**) Chemical structure of RADA. (**c**) Confocal microscopy image of *M. smegmatis* stained with RADA. Adapted from [49] under a Creative Commons license, Elife, 2018.

It is well-established that d-Ala and d-Glu are common amino acids that are present in the peptidoglycan of bacterial cell walls. d-aa are well-known to be incorporated in the peptidoglycan synthesis to build the bacterial cell wall, and their fluorescent derivatives have been proposed for the visualization of the process (Figure 3) [47,48,49].

Interestingly, other d-amino acids can also be produced by bacteria, such as d-Met, d-Leu, d-Phe and d-Tyr, and they have been hypothesized to downregulate peptidoglycan synthesis in adaptation to changes in the surrounding environment [50]. Furthermore, the incorporation of certain d-aa in their cell wall, such as d-Leu, d-Met, d-Trp, and d-Tyr, can inhibit bacterial growth and biofilm formation [51].

## 2. Antimicrobial Peptides (AMPs) with d-Amino Acids

**Figure 4:**
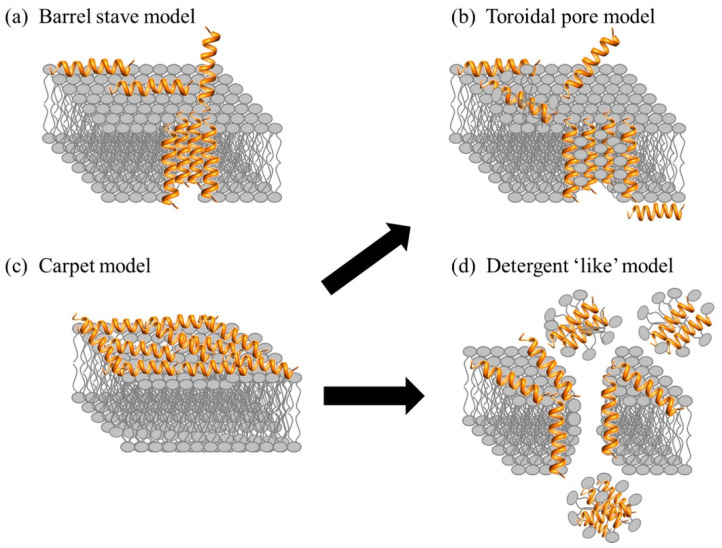
Bacterial cell membrane disruption by AMPs through four common mechanisms. Reproduced from [52] under a Creative Commons license, Biomolecules, 2018.

AMPs are the first line of defence of multi-cellular organisms against pathogenic bacterial infections. They can also be produced by bacteria to gain advantage over other strains that compete for resources in the same niche. AMPs can possess several different conformations and structures, and they are typically amphipathic and often polycationic, so they can electrostatically interact with the polyanionic bacterial membranes. Although many different action mechanisms exist, a large portion of AMPs exert AM activity by disrupting the bacterial cell membrane organization, through four common mechanisms shown in Figure 4 [52].

The introduction of d-aa into the peptide sequence to increase AMP activity is a well-known strategy [53] that helps to improve their pharmacokinetic profile and increases their effectiveness, and it may provide further advantages that still need elucidation [54]. For instance, there is the possibility to access conformational space that is underexplored by natural peptides, which can be advantageous for AMP activity, as is the case of macrocyclic structures, such as cyclosporin and its derivatives [55]. Furthermore, there is an increasing body of evidence that supports the hypothesis that d-aa play an inter-kingdom recognition role at the host–bacteria interface that regulates bacterial colonization and host immune defence [56].

The presence of d-aa in AMPs is attracting increasing interest, as recently reviewed [20]. The earliest findings of their presence in AMPs are as old as 1941, when they were detected in gramicidin and tyrocidine [57], and many more followed, as summarized in Table 1. Several groups arise from *Bacillus* [58] and *Streptomyces* strains [59], and it is well known that Gram-positive bacteria use AMPs, termed bacteriocins [60], as a strategy to strive in an ecological niche with competition from other micro-organisms [61]. Other important sources of AMPs are fungi [62] and frog skin secretions [63]. Although the pursuit of the therapeutic application of many of these peptides had been abandoned over the last decade due to their inherent toxicity [53], we are now witnessing a revival of their use due to an increase in MDR infection occurrence. Today, some AMPs play an important role in the clinic, including colistin, which is considered the last resort against MDR pathogens [64]. In the following sub-sections, we briefly describe the main AMP classes from Table 1, and we refer the readers to existing recent reviews for further details.

### 2.1. Bacitracin

Bacitracin is a natural mixture of cyclopeptides, of which bacitracin A is the most active. This antibiotic displays potent activity on Gram-positive bacteria, which die as a result of cell membrane disruption [92]. Recently, it was found also to be able to neutralize bacterial exotoxins [93]. However, both the narrow spectrum of activity and the high level of nephrotoxicity have significantly restricted its clinical use, so that it is considered as a last-resort treatment [94].

### 2.2. Bombinins

Bombinins have been found only on the skin of the frog species called *Bombina*, from which they derive the name. They are active against both Gram-positive and Gram-negative bacteria, as well as fungi, and they do not lyse erythrocytes, which is a common side effect of AMPs. Conversely, a specific subclass called bombinins H has lower bactericidal activity and can be hemolytic. Interestingly, both L- and D-epimers at the second position have been found in this group, with the latter ones being more active against *Leishmania* parasites [68]. Bombinins’ adoption of amphipathic conformations that mimic the water-membrane interface has been hypothesized to be key in their ability to interact with membranes [95]. The presence of d-*allo*-Ile in bombinin H4 serves as a lipid anchor to enable the formation of a pore in the bacterial membrane, leading to higher activity relative to all L-aa bombinin H2 [96].

### 2.3. Daptomycin

Daptomycin comprises a class of lipid cyclopeptides derived from the soil filamentous bacteria of the genus *Streptomyces*. It is capable of forming transient ionophores in the membrane of target bacteria where it exerts its AMP action [97]. It is effective against drug-resistant Gram-positive bacteria, and for this reason it is applied in the clinic to treat infections of the skin, but also endocarditis associated with methicillin-resistant *S. aureus*. A detailed structure–activity study has demonstrated the importance of several d-aa for AMP activity [69].

### 2.4. Gramicidins

**Figure 5:**
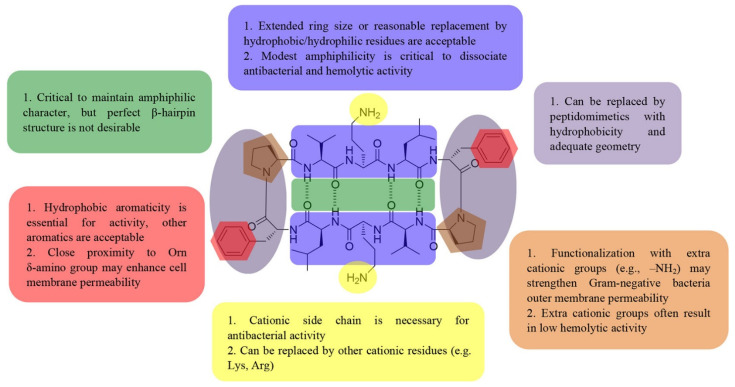
Gramicidin S structure-activity relationship. Reprinted with permission from Ref. [75]. Copyright 2019 American Chemical Society.

Gramicidin is one of the earliest AMPs to be discovered and its main components are gramicidin D and S. The former is a linear peptide that forms homo and hetero dimeric helices that constitute ionic channels in lipid membranes, through which they exert the AM activity. The latter is a cyclopeptide with broader AM activity due to alteration of cell membrane organization. Both types are used to treat topical infections due to their hemolytic effect that prevents systemic applications [98]. To design improved analogues devoid of this side effect, a detailed structure–activity relationship has been delineated throughout the years (Figure 5), from which it has become clear that the β-turn, based on the d-Phe-l-Pro motif, is key for activity, and any modification there requires preservation of the local geometry [75].

### 2.5. Lantibiotics

Lantibiotics are a subclass of bacteriocins which are produced from lactic acid bacteria. They have attracted interest mainly in the food industry as alternatives to synthetic preservatives, which have raised safety concerns [99]. They have attracted interest also as antibiotic substitutes for veterinary use, while their clinical use on humans has been hampered by high production costs, limited stability, and insufficient toxicity studies [100]. However, it is envisaged that innovative formulations may provide a convenient strategy to address at least some of these limitations, especially pertaining to their stability [101]. Furthermore, an attractive feature to pursue their clinical use is that bacteriocins are generally amenable to large-scale green production in bacteria—or even plants—through the use of biotechnology [102].

### 2.6. Polymyxins

Polymyxins are amongst the earliest AMPs to be discovered in the 1940s. They were approved for clinical use in late 1950s and abandoned soon after due to their nephrotoxicity. Recently, their use has been revived as a last-resort treatment against MDR pathogens [103]. Elucidating their structure–activity relationship has thus become crucial to develop new derivatives with improved safety profiles [64]. Polymyxins exert their primary AM activity through direct interaction with the lipid A component of the lipopolysaccharide (LPS), which then leads to the disruption of its function as a physical barrier. However, they also have secondary modes of action that are under elucidation [104]. The recently discovered resistance to polymyxins has prompted their use in combination therapy with antibiotics, although this practice remains highly debated [105]. Their poor permeability and low absorption in the gastrointestinal tract have prompted research on innovative delivery systems to overcome these limitations [106].

### 2.7. Streptogramins

Streptogramins comprise two compounds corresponding to type A and type B, which inhibit bacterial protein synthesis and thus exert bactericidal action on Gram-positive bacteria, including MDR strains. Although various mechanisms of AMR have been identified in cocci, its occurrence in clinical isolates fortunately remains very low. They are effective in treating severe infections caused by Gram-positive bacteria, however, their clinical use remains limited, mainly due to adverse reactions [107]. Surprisingly, the complete characterization of streptogramin B by ^1^H and ^13^C nuclear magnetic resonance (NMR) spectroscopy was only recently reported [84], despite the fact that it has long been known for its AMP activity. A modular and scalable synthesis of type A compounds was recently reported to enable structural modifications that could address the poor physicochemical properties that limit their clinical use [83].

### 2.8. Vancomycin

Vancomycin is a glycopeptide that for decades has been considered the last resort treatment against infections determined by Gram-positive bacteria. However, the emergence of vancomycin-resistant (VR) strains, especially *S. aureus* (VRSA), have raised great health concerns, and the urgent search for new AMPs that can provide effective treatment [108] is currently underway. Chemists have made great efforts to provide improved synthetic protocols to access vancomycin-related structures [91]. One promising approach to counteract AMR is modification to include lipophilic membrane anchors and cell-penetrating cationic peptides [109].

## 3. Polymer-Conjugates of AMPs with d-aa

**Figure 6:**
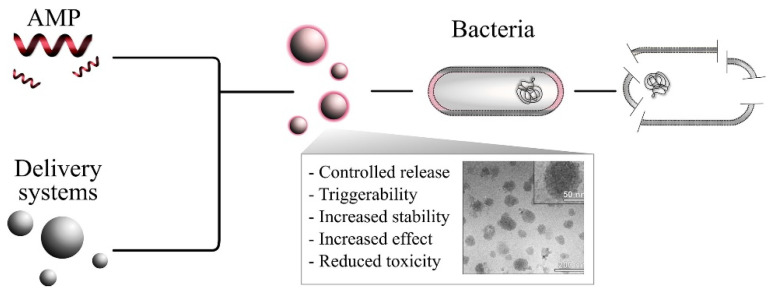
The development of advanced delivery systems for AMPs can be advantageous. Reprinted with permission from Ref. [124]. Copyright 2017 Elsevier.

The therapeutic application of AMPs poses many challenges, including high production costs, the risk of adverse effects, and a typically short half-life due to rapid enzymatic degradation. The mitigation of these risks is possible through the use of polymers, for instance poly-(α-amino acid)-structures that mimic AMPs [110], or other types of polymers with AM activity, which are highly researched [111,112,113], in addition to dendrimers [114,115,116]. The AMP-mimetic design deserves a separate discussion and interested readers can find further details in the recent literature [117,118,119,120,121,122,123]. More generally, the development of advanced delivery systems has been proposed as a convenient strategy to enable the therapeutic translation of AMPs (Figure 6), although the understanding of AMP-carrier interactions and their effects on release and activity is a complex matter that requires thorough elucidation [124]. Considering that AMPs often display a polycationic nature, their complexation with polyelectrolytes is an attractive avenue for their formulation [125]. In recent years, there has been increasing interest in the development of various vehicles for AMP delivery [126], such as vesicles [127], microgels and hydrogels [128], natural fibres [129], and nanostructured systems [130], including electro-spun fibres [131,132], in addition to many others [133,134,135,136]. Indeed, working on a nanoscale offers further advantages in medicinal chemistry, both from a qualitative and a quantitative point of view [137]. In particular, the supramolecular assemblies of polymers and AMPs are a hot topic that has been recently reviewed and, thus, will not be discussed here [138].

Here, the focus will be on recent examples of polymer-AMP covalent conjugates that include d-aa. The synthetic approaches to obtain them will not be discussed, since they have recently been reviewed elsewhere, in addition to the various types of polymeric structures [139,140]. Instead, we will discuss the recent progress over the last five years pertaining to polymer conjugates with the AMPs shown in Table 1.

### 3.1. Bacitracin-Polymer Conjugates

**Figure 7:**
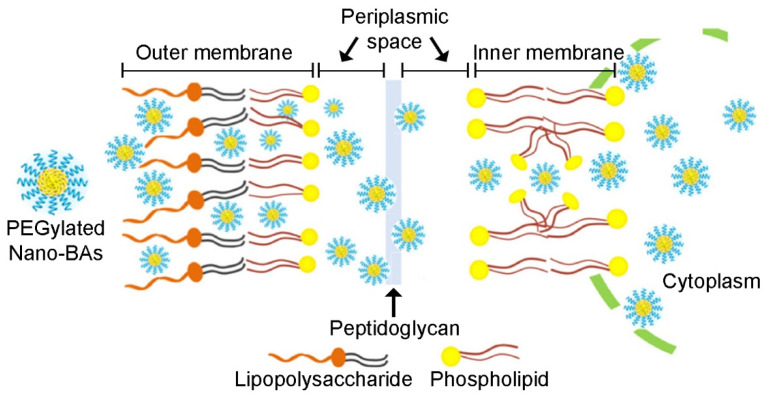
Proposed mechanism of action on Gram-negative bacteria of bacitracin-polymer nanosized micelles (PEGylated Nano-BAs) through LPS interaction. Reprinted with permission from Ref. [142]. Copyright 2018 American Chemical Society.

The conjugation of bacitracin A with poly(d,l-lactic-*co*-glycolic acid) (PLGA) enables self-assembly into nanosized micelles, which display broader and stronger activity, and higher biocompatibility, especially with longer polymer chains, which have, unfortunately, significantly limited water solubility [141]. The addition of more hydrophilic polyethylene glycol (PEG) to yield PEG-PLGA-PEG triblock copolymers proved to be an effective strategy to solve this issue, whilst preserving the ability to self-assemble into micelles (Figure 7). The resulting nanoparticles displayed activity against both Gram-positive and Gram-negative bacteria. In the latter case, interaction with lipopolysaccharide (LPS) is likely to lead to membrane depolarization and subsequent disruption. Accumulation in inflammatory tissue and long circulation times also enables the treatment of thigh infections in vivo in mouse models [142]. These micelles were found to be effective against penicillin-resistant *S. pneumoniae* strains [143]. Furthermore, the same type of approach was demonstrated using Pluronic^®^ polymers F127, P123 and P85, of which the latter was more efficient in vivo too, without significant toxicity being noted in major organs [144].

### 3.2. Daptomycin-Polymer Conjugates

Daptomycin has been linked to a poly-amine siderophore to enable activity against carbapenem-resistant Gram-negatives, which use this type of compound to sequester iron through an active transport process that is important for bacterial growth and virulence [145]. Although the siderophore used in this study is not a polymer, the strategy to employ formation of an amide bond for conjugation could also be potentially applied to macromolecules.

**Figure 8:**
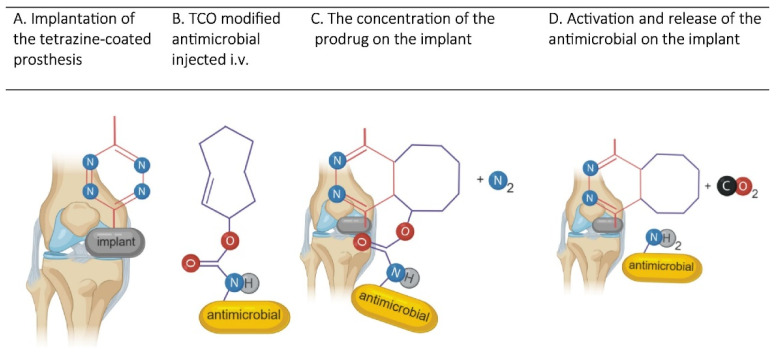
Daptomycin conjugation to a medical-implant coating through bio-orthogonal click chemistry based on tetrazine and *trans*-cyclooctene (TCO). Reprinted with permission from Ref. [146]. Copyright 2020 Elsevier.

In another study, a mussel-inspired catechol-based adhesive polymer was envisaged to coat titanium implants. Coupling of the macromolecule with tetrazine was then used as a strategy to enable bio-orthogonal click chemistry for the anchoring of daptomycin (Figure 8). In particular, this AMP was bound to trans-cyclooctene to obtain an inactive prodrug that could undergo an inverse electron demand Diels–Alder reaction and allow its conjugation as a prodrug, for the subsequent release upon hydrolysis of a carbamate functionality [146].

### 3.3. Gramicidins

In 2020, an innovative approach was reported for the rapid covalent binding—in water and at room temperature—of biomolecules bearing primary amines, such as gramicidin S on pre-formed polymers. The strategy could be applied both to reversible addition-fragmentation chain transfer (RAFT) and to atom-transfer radical polymerization (ATRP), which are modern methods to exert fine control over the final polymer molecular weight distribution. In particular, the use of a trifluoroborate iminium functionality on the monomers enabled its quantitative conversion into potassium acyltrifluoroborates (KATs) after polymerization. KAT moieties then reacted with either one of two AMP analogues through amide bond formation. In particular, the AMP was first derivatized on its ornithine amino sidechain with either one of two hydroxylamine linkers, of which one was photocleavable. The orthogonal ligation between hydroxylamines and KAT-modified polymers is highly chemo-selective, and subsequent biological tests indeed confirmed that the AMP activity could be restored through a UV-triggered release from the polymer [147].

### 3.4. Polymyxins

Colistin has been conjugated to dextran through the use of a linker to provide conjugates with tuneable molecular weight and physico-chemical properties, depending on the type of dextran used, and on its chemical modifications. The conjugates can accumulate in infected wounds where amylase is more abundant, relative to human serum, so that enzymatic hydrolysis releases the active drug. However, this approach is not trivial, as spectroscopic analysis revealed the presence of residual AMP bound to the linker, which affects its bioactivity [148].

A different approach was used to link colistin to a poly(ethylene glycol) methyl ether acrylate (PEGA) polymer, which has attracted attention as a preferable alternative to high molecular weight PEG. In this case, colistin was first protected on its amino functions, and then the hydroxyl groups of its two Thr sidechains were esterified with an acid linker, providing a hydrolytically labile α-halo ester moiety. Next, a “grafting from” approach allowed the generation of the macromolecular product via copper-mediated photoinduced living radical polymerization (CP-LRP). Subsequent experiments confirmed that AMP activity was preserved, and the AMP structure did not undergo undesired chemical modifications [149].

**Figure 9:**
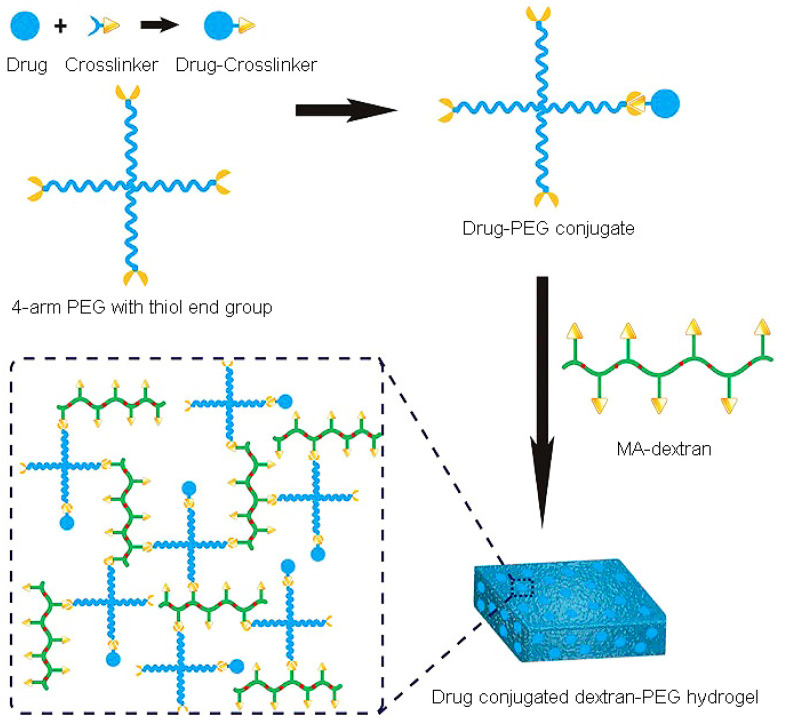
Covalent linkage strategy to bind polymyxin B or vancomycin to a linker via amide bonds, and then to a 4-arm PEG with terminal thiol groups, to allow subsequent linkage to a methacrylate(MA)-dextran polymer. Reprinted with permission from Ref. [150]. Copyright 2017 WILEY-VCH Verlag GmbH & Co. KGaA, Weinheim.

Finally, both dextran and PEG were employed to covalently bind polymyxin B and vancomycin, so as to provide a wound-dressing hydrogel able to eradicate bacterial infections and inhibit further microbial growth (Figure 9). In this case, amine groups of both AMPs were reacted with the orthogonal BMPS linker, which features a *N*-hydroxysuccinimide ester moiety on one end and a maleimido on the other. In this manner, the drug-linker conjugates could be bound to 4-armed PEG chains ending with thiol groups. The unreacted thiols were then bound to methacrylate-dextran to yield the final gel. Interestingly, both AMPs’ activity was preserved and prevention of their release from the wound dressing thanks to covalent binding to the dual polymer was envisaged as a convenient strategy to avoid systemic side effects [150].

### 3.5. Vancomycin

Vancomycin covalent conjugation to a polymer without significant loss of AM activity is not at all trivial. Indeed, this was the case even when using cationic polymers with inherent AM activity. The two components were bound to each other through a PEG diacrylate linker to undergo a Michael addition to the AMP on one side, and cross-metathesis to the cationic polymer on the other. The conjugates displayed significant loss of AM activity relative to the AMP and the cationic polymer alone, although the presence of the PEG linker appeared to enhance the biocompatibility profile in vitro [151].

However, AM activity can be displayed after polymer conjugation. In one study that aimed at addressing the problem of infections occurring on titanium-based orthopaedic implants, azido-functionalized methacrylate chains were grafted from a titanium alloy to enable subsequent orthogonal click chemistry with alkynylated vancomycin. The AMP-coated surfaces were less susceptible to *S. aureus* adhesion and colonization, both in vitro and in vivo [152].

Vancomycin-polymer conjugates have also been employed for diagnostics. In this case, both branched and linear polymers were prepared from *N*-isopropyl acrylamide monomers, various linkers, Nile Red dye for detection, and vancomycin to be conjugated through the amino groups. Fluorescence and calorimetric data indicated that the branched polymer was more effective in binding both d-Ala-d-Ala as a model target, and whole Gram-positive bacteria [153]. These results confirmed earlier findings pertaining the better performance of branched polymers relative to linear analogues, and the requirement of having the AMP displayed at the chain ends for target recognition, and to enable the polymer coil-to-globule transition in doing so for detection [154].

## 4. Conclusions and Future Perspectives

In recent years, we have witnessed a revival of AMPs for clinical use, although many challenges in terms of side effects and resistance emergence are yet to be completely solved. The introduction of d-aa in the AMPs sequence represents an attractive approach aimed to improve both their activity and their metabolic stability. Non-proteogenic d-aa are widespread throughout biological systems, including microbes and metazoan organisms, where they make up the structural components and are involved in the regulation of different functions. Furthermore, it is worth noting that using non-proteogenic d-aa often raises toxicity concerns for clinical applications, despite the very promising results of in vivo studies [142], with no significant toxicity being observed in major organs [144].

Covalent conjugation with polymers is an attractive approach to modulate the pharmacokinetic profile of AMPs, especially, to provide AM coatings for medical implants and devices, although their effective design is far more trivial, since AM activity loss may arise from the covalent linkages to AMPs. Future opportunities may arise also from combination strategies that employ organic and inorganic components [155], especially if taking advantage of nanotechnology [156,157].

In particular, there is an outstanding need for the development of AM coatings for medical implants and devices [158]. To this end, a vivid research area concerns the development of macromolecules that are capable of avoiding the formation of biofilms [159], which are particularly challenging to eradicate [160]. Current antibiotic therapies are simply insufficient to address the insurgence of local infections in their immediate surroundings, especially in the long-term [161]. In addition to orthopaedics, dental healthcare [162], cardiac [163] and urological applications [164] are highly sought after. Another growing area of research involves food active packaging [165].

An alternative approach is the use of supramolecular polymers that are dynamic in nature and could thus respond to various stimuli as needed [166,167,168]. In particular, self-assembling short peptides with inherent AM activity residing only in their assemblies are particularly attractive as economical smart materials, enabling the switching on/off of AMP activity through assembly/disassembly cycles as desired [169,170]. Molecules as simple as amino acids [171,172,173] or dipeptides [174] were modified to display hydrophobic groups facilitating self-association in water to form AM hydrogels, demonstrating that this approach, in line of principle, is feasible. Clearly, mastering the behaviour of dynamic supramolecular systems in vivo poses an additional level of challenges to overcome, but also the potential to provide innovative solutions to unsolved clinical problems.

## Figures and Tables

**Table 1:** Natural AMPs with d-aa.

Peptide Class	Origin	Type	d-aa	Ref.
Actinomycins	Bacteria	Cyclodepsipeptides	d-Val	[65]
Alternaramide	Fungi	Cyclodepsipeptide	d-Phe	[66]
Bacitracins	Bacteria	Cyclopeptides	d-Asp d-Glu d-Orn d-Phe	[67]
Bassianolide	Fungi	Cyclodepsipeptide	d-hydroxyvaleric acid	[66]
Bombinins	Frog	α-helical linear peptides	d-Ile d-Leu	[68]
Daptomycins	Bacteria	Cyclolipodepsipeptides	d-Ala d-Asn d-Ser	[69]
Etamycin-type, Fijimycins	Bacteria	Cyclopeptides	d-Ala d-Leu d-Hyp d-Thr d-Ser	[70,71]
Fengycin	Bacteria	Cyclolipopeptide	d-Ala d-*allo*-Thr d-Orn d-Tyr	[72]
Fusaricidins	Bacteria	Cyclolipodepsipeptides	d-Ala d-*allo*-Thr	[73]
Gramicidin D	Bacteria	Helical peptides	d-Leu d-Val	[74]
Gramicidin S	Bacteria	Cyclopeptides	d-Phe	[75]
Hasadillins	Bacteria	Cycloglycolipopeptides	d-*allo*-Thr d-Glu d-Thr d-Tyr	[76]
Himastatin	Bacteria	Cyclodepsipeptide	d-Thr d-Val	[66,77]
Iturins	Bacteria	Cyclolipopeptides	d-Asn d-Ser d-Tyr	[78]
Lantibiotics	Bacteria	Linear/Cyclic peptides	d-Ala d-aminobutyrate S-[(*Z*)-2-aminovinyl]-d-Cys	[61]
Monamycins	Bacteria	Cyclodepsipeptides	d-Ile d-Val *N*-Methyl-d-Leu	[66,79]
Mycobacillin	Bacteria	Cyclopeptides	d-Asp d-Glu	[80]
Peacilodepsipeptide A	Fungi	Cyclodepsipeptide	d-Ala d-Tyr	[81]
Polymyxins	Bacteria	Cyclolipopeptides	d-Leu d-Phe d-Ser	[64]
Pullularins	Fungi	Cyclodepsipeptides	d-3-phenyllactic acid	[82]
Ramoplanins	Bacteria	Cyclodepsipeptides	d-Ala d-*allo*-Thr d-hydroxyphenyl-Gly d-Orn	[73]
Streptogramins A	Bacteria	Cyclopeptides	d-Pro	[83]
Streptogramins B	Bacteria	Cyclodepsipeptides	d-Aminobutyrric acid	[84]
Surfactins	Bacteria	Cyclolipopeptides	d-Leu	[85]
Teicoplanin	Bacteria	Cycloglycopeptide	d-*m*-chloro-β-hydroxy-Tyr d-*p*-hydroxyphenyl-Gly	[86]
Tolaasins	Bacteria	Cyclolipodepsipeptides	d-*allo*-Thr d-2,4-diaminobutyric acid d-homoserine d-Gln d-Leu d-Pro d-Ser d-Val	[87,88]
Tyrocidines	Bacteria	Cyclopeptides	d-Phe d-Trp	[89]
Valinomycins	Bacteria	Cyclodepsipeptide	d-Val d-hydroxy-*iso*-Val	[90]
Vancomycin	Bacteria	Cycloglycopeptide	d-*m*-chloro-β-hydroxy-Tyr d-*p*-hydroxyphenyl-Gly d-Leu	[91]
